# Classification Models for COVID-19 Test Prioritization in Brazil: Machine Learning Approach

**DOI:** 10.2196/27293

**Published:** 2021-04-08

**Authors:** Íris Viana dos Santos Santana, Andressa CM da Silveira, Álvaro Sobrinho, Lenardo Chaves e Silva, Leandro Dias da Silva, Danilo F S Santos, Edmar C Gurjão, Angelo Perkusich

**Affiliations:** 1 Federal University of the Agreste of Pernambuco Garanhuns Brazil; 2 Federal University of Campina Grande Campina Grande Brazil; 3 Federal University of Alagoas Maceió Brazil; 4 Federal Rural University of the Semi-Arid Pau dos Ferros Brazil

**Keywords:** COVID-19, test prioritization, classification models, medical diagnosis

## Abstract

**Background:**

Controlling the COVID-19 outbreak in Brazil is a challenge due to the population’s size and urban density, inefficient maintenance of social distancing and testing strategies, and limited availability of testing resources.

**Objective:**

The purpose of this study is to effectively prioritize patients who are symptomatic for testing to assist early COVID-19 detection in Brazil, addressing problems related to inefficient testing and control strategies.

**Methods:**

Raw data from 55,676 Brazilians were preprocessed, and the chi-square test was used to confirm the relevance of the following features: *gender*, *health professional*, *fever*, *sore throat*, *dyspnea*, *olfactory disorders*, *cough*, *coryza*, *taste disorders*, and *headache*. Classification models were implemented relying on preprocessed data sets; supervised learning; and the algorithms multilayer perceptron (MLP), gradient boosting machine (GBM), decision tree (DT), random forest (RF), extreme gradient boosting (XGBoost), k-nearest neighbors (KNN), support vector machine (SVM), and logistic regression (LR). The models’ performances were analyzed using 10-fold cross-validation, classification metrics, and the Friedman and Nemenyi statistical tests. The permutation feature importance method was applied for ranking the features used by the classification models with the highest performances.

**Results:**

*Gender*, *fever*, and *dyspnea* were among the highest-ranked features used by the classification models. The comparative analysis presents MLP, GBM, DT, RF, XGBoost, and SVM as the highest performance models with similar results. KNN and LR were outperformed by the other algorithms. Applying the easy interpretability as an additional comparison criterion, the DT was considered the most suitable model.

**Conclusions:**

The DT classification model can effectively (with a mean accuracy≥89.12%) assist COVID-19 test prioritization in Brazil. The model can be applied to recommend the prioritizing of a patient who is symptomatic for COVID-19 testing.

## Introduction

### Overview

In modern medical systems, health care professionals, managers, and governments use information and data analysis to make decisions [1]. Data is stored, enabling rapid access and sharing during the diagnosis, monitoring, and treatment of patients. Therefore, there are propositions of eHealth and mobile health (mHealth) systems to assist health care professionals and policy makers with decision making [2,3]. Such systems are relevant to provide decision support advice based on patients’ data, helping health care professionals and policy makers address problems related to inefficient COVID-19 testing and control strategies (eg, limited testing resources) in low- and middle–income countries [4]. For example, people who live in low- and middle–income settings, remote settings, and hard-to-reach settings are the most affected by precarious health care. Such a situation is even more critical in a pandemic scenario.

COVID-19 is a disease caused by SARS-CoV-2 [5]. In December 2019, the first cases of COVID-19 appeared in Wuhan, Hubei Province, China [6]. Due to the high growth of COVID-19 confirmed cases worldwide, on January 30, 2020, the World Health Organization considered the COVID-19 outbreak a Public Health Emergency of International Concern [7].

### Motivation and Problem Statement

As the number of COVID-19 confirmed cases continuously increases, health care professionals and policy makers need to define guidelines to prevent the disease, delaying the transmission rates. Such guidelines are relevant due to the high probability of collapse in health services and shortages of medical supplies (eg, testing resources) [8]. Confirmation of the first COVID-19 case in Brazil was in March 2020, and since then, there has been an upward trend in confirmed cases and deaths. Unfortunately, the Brazilian government has reported more than 13 million cases, with more than 333,000 deaths. Currently, Brazil is one of the most affected countries by COVID-19, with insufficient control measure implementation. Controlling the COVID-19 outbreak in Brazil is a challenge of continental proportions due to the population’s size and urban density, inefficient maintenance of social distancing and testing strategies, and limited availability of testing resources [9].

This study addresses the COVID-19 testing prioritization for patients who are symptomatic to assist early COVID-19 detection in Brazil. Addressing this problem is relevant due to the need for prioritization guidelines to improve testing and control strategies’ efficiency. Therefore, the main research question (RQ) is can demographic characteristics and symptoms that do not require expensive exams effectively assist the test prioritization for early COVID-19 detection in Brazil? From the main RQ, the four secondary RQs are (1) what demographic characteristics are relevant to conduct the test prioritization? (2) what symptoms are suitable to drive the test prioritization? (3) what is the most suitable classification model for test prioritization? and (4) what are the impacts of the reduction of reported symptoms in the test prioritization?

### Aim of the Study

The study relied on preprocessing a raw data set with information on 55,676 patients, aiming to provide a classification model that effectively recommends or not the prioritization of patients who are symptomatic for COVID-19 testing (ie, a binary classification problem). The implementation of classification models also relied on supervised learning and the algorithms multilayer perceptron (MLP), gradient boosting machine (GBM), decision tree (DT), random forest (RF), extreme gradient boosting (XGBoost), k-nearest neighbors (KNN), support vector machine (SVM), and logistic regression (LR). The algorithms were trained and tested using preprocessed data sets composed of demographic characteristics and reported symptoms that do not require expensive exams [10]. Use of such symptoms is a relevant strategy for COVID-19 test prioritization due to the majority of the Brazilian population’s high poverty levels [11].

Our findings also provide insights for developers of eHealth and mHealth systems when choosing the most suitable classification model for COVID-19 testing prioritization. Such insights are also relevant for health care professionals and policy makers who envision applying a classification model to prioritize patients who are symptomatic for testing. The study enhances the state of the art by providing three main contributions: (1) the preprocessing of raw data from 55,676 Brazilians, with the availability of data related to patients who are symptomatic [10]; (2) the implementation of classification models, along with reports of feature ranking, to support COVID-19 test prioritization [12]; and (3) a comparative analysis of the classification models.

## Methods

### Overview

**Figure 1:**
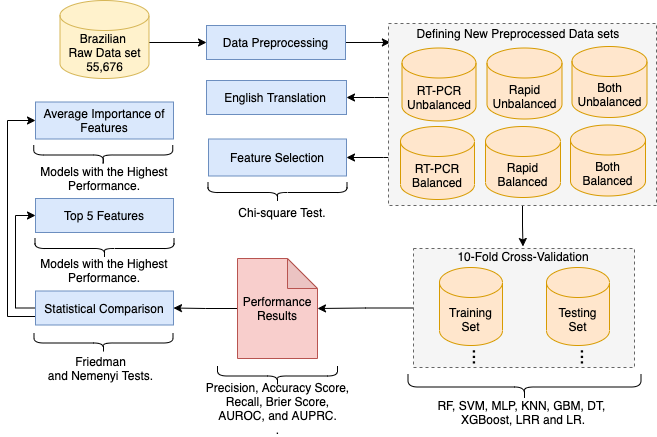
Overview of the research methodology applied for the study. The methodological steps consist of data preprocessing, the definition of new data sets, English translation, feature selection, 10-fold cross-validation, statistical comparisons, and feature ranking. AUPR: area under the precision-recall curve; AUROC: area under the receiver operating characteristic curve; DT: decision tree; GBM: gradient boosting machine; KNN: k-nearest neighbors; LR: logistic regression (weak regularization); LRR: logistic regression (strong regularization); MLP: multilayer perceptron; RF: random forest; RT-PCR: reverse transcription polymerase chain reaction; SVM: support vector machine; XGBoost: extreme gradient boosting.

This study’s research methodology consists of data preprocessing, the definition of new data sets, English translation, feature selection, 10-fold cross-validation, statistical comparisons, and feature ranking (Figure 1). The raw data from 55,676 Brazilians were preprocessed to define new data sets with information about patients who are symptomatic tested for COVID-19 using reverse transcriptase polymerase chain reaction (RT-PCR) and rapid tests (antibody and antigen). The textual descriptions of six preprocessed data sets (ie, *RT-PCR unbalanced*, *RT-PCR balanced*, *rapid unbalanced*, *rapid balanced*, *both unbalanced*, and *both balanced*) were translated from Portuguese into English for public data availability. The chi-square test was applied in the new data sets to support the feature selection with a *P*<.01, verifying the relevance of features for the classification task by dependence and independence relations [13]. The chi-square test for independence compared two variables in a contingency table to verify if they relate to each other.

We applied the 10-fold cross-validation method, with five repetitions, to validate the MLP, GBM, DT, RF, XGBoost, KNN, SVM, and LR (weak/strong regularization) classification models using the six data sets. We selected such algorithms because they have different characteristics such as using neural layers, tree combinations, and calculating the distance between data. The mean results for classification metrics were also calculated: precision, accuracy score, recall, Brier Score, area under the receiver operating characteristic curve (AUROC), and area under the precision-recall curve (AUPRC). The recall results were further analyzed using the Friedman and Nemenyi statistical tests to improve the classification models’ comparisons. We used the Friedman test to verify the differences between classification models. We applied the Nemenyi test to group classification models based on the verification of differences using multiple comparisons. Finally, we conducted features’ ranking for each classification model with the highest performance using the permutation feature importance method, providing average importance and SD. The source code for replication is available in a GitHub repository [12].

### Data Collection

The raw data from 55,676 Brazilians included information on tested patients in a spreadsheet format. However, the data collection is not a contribution of this study. The raw data was collected by the public health agency of the city of Campina Grande, Paraíba State in Northeast Brazil. Such a public agency is informed by all the COVID-19 exams performed in the city of Campina Grande. The health agency employees removed patient identification, and the data made available were reused to enable this study. The raw data set comprises categorical features such as *health professional*, *security professional*, *ethnicity*, *test type*, *fever*, *sore throat*, *dyspnea*, *olfactory disorders*, *cough*, *coryza*, *taste disorders*, *headache*, *additional symptoms*, *test result*, *comorbidities*, *test status*, and *symptoms description*.

### Data Preprocessing

We conducted the data preprocessing using the Python programming language. The raw data set was preprocessed by applying string matching algorithms to correct inconsistencies. One example of inconsistency was the occurrence of empty columns of symptoms; however, the same symptoms were in a column for the general description of symptoms.

Furthermore, the following instances from the total 55,676 sample were removed due to our exclusion criteria: patients with uncompleted tests or undefined final classifications (n=12,929, 23.22%), duplicated instances (n=251, 0.45%), outliers related to input errors (n=10,408, 18.69%), test types that are not RT-PCR or rapid (n=771, 1.38%), undefined gender (n=27, 0.05%), and patients who were asymptomatic (n=11,269, 20.24%). Patients who were asymptomatic were removed because the inputs for the algorithms rely on demographic characteristics and symptoms.

Removing the feature related to the symptoms’ descriptions provides dimensionality reduction in the raw data set feature space. For example, fatigue was removed because the symptom was reported by 228 (0.41%) of the 55,676 patients. Given the main focus on symptoms, the data sets did not include comorbidities and the remaining features (eg, *ethnicity*). As inclusion criteria, the most frequently reported symptoms (ie, fever, sore throat, dyspnea, olfactory disorders, cough, coryza, taste disorders, and headache) and relevant demographic characteristics (ie, gender and health professional) were selected as features of unbalanced and balanced data sets (Table 1). Health care professionals were considered relevant due to the frequency of exposure to SARS-CoV-2. However, for gender, there is no consensus if there is a difference in the proportions of males and females infected with SARS-CoV-2 (usually a relatively even distribution) [14,15].

**Table 1:** Demographic and symptoms from patients who are symptomatic of both test type data sets.

Features			Unbalanced (n=20,021)		Balanced (n=3128)
**Demographic characteristics**					
	Gender: male, n (%)	8919 (44.55)		1639 (52.40)	
	Health professional, n (%)	2485 (12.41)		475 (15.19)	
**Symptoms**					
	Fever, n (%)	9169 (45.80)		1856 (59.34)	
	Sore throat, n (%)	5976 (29.85)		848 (27.11)	
	Dyspnea, n (%)	3704 (18.50)		1082 (34.59)	
	Olfactory disorders, n (%)	1967 (9.82)		522 (16.69)	
	Cough, n (%)	11,641 (58.14)		1944 (62.15)	
	Coryza, n (%)	1159 (5.79)		266 (8.50)	
	Taste disorders, n (%)	1596 (12.37)		387 (12.37)	
	Headache, n (%)	4034 (20.15)		577 (18.45)	

The categorical data were converted into binary representations during the preprocessing. For the feature *gender*, the number 0 represents a female patient, and 1 represents a male. For the features *health professional*, *fever*, *sore throat*, *dyspnea*, *olfactory disorders*, *cough*, *coryza*, *taste disorders*, and *headache*, the number 0 represents a positive response, and 1 represents a negative response. For each data set, the *test result* was the class that can be labeled as 0 for recommending a patient who is symptomatic for COVID-19 test prioritization or 1 for not recommending such patient’s prioritization.

The preprocessing included undersampling using the near-miss technique [16], considering COVID-19 positive and negative cases. Undersampling was applied instead of oversampling to prevent the use of synthetic data in training and testing sets. However, as stated, unbalanced data were also considered, without undersampling, to improve the experiments’ representativity and to achieve a scenario closer to a real-world setting, with more negative than positive COVID-19 cases.

**Figure 2:**
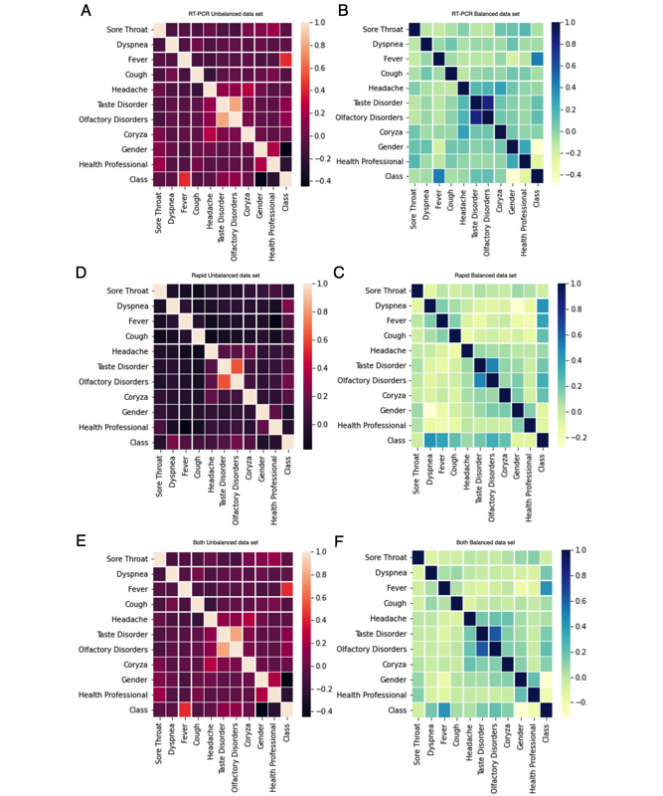
Correlation matrix for (A) RT-PCR unbalanced data set, (B) RT-PCR balanced data set, (C) rapid unbalanced data set, (D) rapid balanced data set, (E) both unbalanced data set, and (F) both balanced data set. RT-PCR: reverse transcription polymerase chain reaction.

Using the chi-square test for the *both unbalanced* and *both balanced* data sets, the independence hypothesis was only confirmed for *headache*. For the *RT-PCR unbalanced* data set, the independence hypothesis was confirmed for *sore throat*, *dyspnea*, *headache*, and *coryza*. In the *rapid unbalanced* data set, the independence hypothesis was confirmed for *sore throat* and *health professionals* features. For the *RT-PCR balanced* data set, the independence hypothesis was confirmed for *dyspnea*, *cough*, *headache*, and *coryza*; while for the *rapid balanced* data set, the hypothesis was only confirmed for *sore throat*. Such information was used for feature selection during the experiments, presenting scenarios with different numbers of symptoms to implement classification models. Furthermore, we used a correlation matrix to analyze the correlation coefficients between the features for each data set (Figure 2). For example, *fever* was among the features with the highest correlation coefficients for all data sets.

**Figure 3:**
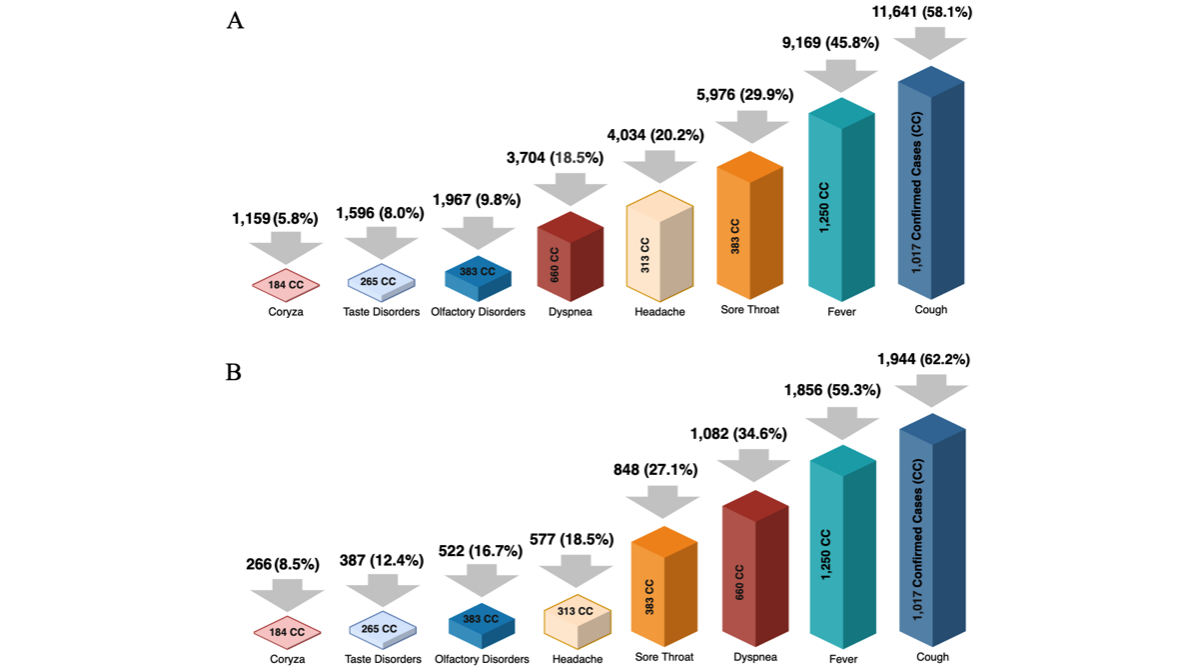
(A) The frequency of symptoms for the 20,021 patients who were symptomatic of the both unbalanced data set and the number of CCs. Top values are frequencies; numbers on the geometric forms are the CC for frequency. (B) The frequency of symptoms for the 3128 patients who were symptomatic of the both balanced data set and the number of CCs.

The *both unbalanced* data set was composed of 20,021 patients tested by both RT-PCR and rapid tests. The reduction in the number of patients occurred due to the uncompleted tests, duplicated instances, outliers related to input errors, test type, and patients who were asymptomatic. The *both unbalanced* data set contained 1564 (7.81%) positive and 18,457 (92.19%) negative COVID-19 cases, while the balanced one included 1564 cases of each class. From the female patients, 496 (2.48%) were positive and 10,606 (52.97%) were negative cases. For male patients, 1068 (5.33%) were positive and 7851 (39.21%) were negative cases. Cough was the most frequent symptom (n=11,641, 58.1%). Fever was the second most common symptom (n=9169, 45.8%). The remaining symptoms were reported by at most 5976 (29.9%) patients who were symptomatic (Figure 3A).

The *both balanced* data set contained 3128 patients tested by RT-PCR and rapid tests. The near-miss technique reduced the number of negative cases to be equal to positive cases; 496 (15.86%) were positive and 993 (31.75%) were negative cases from the female patients. For males, 1068 (34.14%) were positive and 571 (18.25%) were negative cases. Cough and fever continued to be the first and second most frequently reported symptoms, respectively. The remaining symptoms were also reported by at most 1082 (34.6%) patients (Figure 3B).

Finally, the *RT-PCR unbalanced* data set included 916 (32.96%) positive and 1863 (67.04%) negative COVID-19 cases, while the balanced one included 916 cases of each class. The *rapid unbalanced* data set included 648 (3.76%) positive and 16,594 (96.24%) negative COVID-19 cases, while the balanced one included 648 of each class. The six scenarios’ presentations aim to compare the classification models’ results using various test types. Thus, there was no requirement to implement different clinical protocols or select patients with specific profiles for testing based on the results related to the six scenarios presented in this paper.

### Algorithms

We implemented the classification models using supervised learning and the MLP, GBM, DT, RF, XGBoost, KNN, SVM, and LR algorithms. An MLP machine learning (ML) algorithm [17] of one hidden layer learns the function:


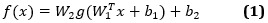


where *W*_1_ represents the weights of the input layer, *W*_2_ represents the hidden layer, *b*_1_ is the bias added to the hidden layer, *b*_2_ is the output layer, and *g* is the activation function.

The GBM is a fixed-size DT that uses a boosting strategy [18]. This ML algorithm has a built-in feature selection and aims to provide the estimation or approximation 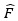 or the function *F* * (*x*) that maps *x* to *y*, minimizing the expected value using a loss function *L*(*y*, *F*(*x*)) over the joint distribution [19], given by:

*F* * = *arg min_F_E_y_*_,_*_x_L*(*y*,*F*(*x*)) = *arg min_F_E_x_*[*E_y_*(*y*,*F*(*x*)) | *x*] **(2)**

A DT is an ML algorithm that usually uses a divide and conquer strategy to generate a directed acyclic graph by applying division rules based on information gain [20]. The algorithm has a built-in feature selection, and the information gain is guided by the concept of entropy *H*, which measures the randomness of a discrete random variable *A* (with domain *a*_1_, *a*_2_,..., *a_n_*), given by:


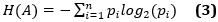


where *p_i_* is the probability of observing each value *a*_1_, *a*_2_,..., *a_n_*. This algorithm enables a straightforward interpretation of results by following the decision rules of a unique tree.

The RF is an ML algorithm that relies on classification and regression trees, following specific tree growing rules, tree combination, self-testing, and postprocessing [21]. The algorithm has a built-in feature selection, assessed by the Gini impurity criterion index. The binary split of a node *n* is given by:


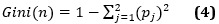


where *p_j_* is the relative frequency of class *j*. This algorithm also enables a straightforward interpretation of results by following the decision rules of the trees.

As a variant of the GBM, the XGBoost is a regression tree with the same decision rules as a DT [22]. If the XGBoost ML algorithm consists of *K* DTs, the optimization objective function is given by:


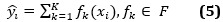


where *f_k_* is an independent tree with leaf scores, and *F* is the space of a regression tree. Both algorithms enable a straightforward interpretation of results.

The KNN is a distance-based ML algorithm that identifies a new instance based on a neighbor’s distance [23]. An instance represents a point in the space, and the algorithm calculates the distance between two points using a metric such as the Euclidean distance, given by:


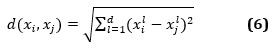


where *x_i_* and *x_j_* are vectors representing objects in the space, and 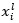 and 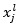 are the *l-th* elements of the vectors.

The SVM is a ML algorithm that handles binary data using a line to achieve the maximum distance between the data. The algorithm comprises four basic concepts: separation hyperplane, maximum margin hyperplane, soft margin, and kernel function [17]. For instance, the maximization of the margin hyperplane is given by:


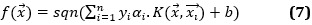


where *y_i_* are the output variables, *x_i_* are input vectors, *b* is the bias, *K* is a dot products function (Kernel), and *α_i_* is calculated by the maximization of:


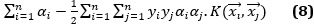


where *x_j_* are the named support vectors when *α_i_* is greater than 0.

Finally, the LR is an extension of linear regression that estimates relations between variables using a sigmoid function during probabilistic classifications [24], given by:


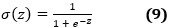


where *z* is the weighted sum of the evidence of a class. Regularization can also be used to prevent overfitting. We applied the LR algorithm to compare a compact and linear model’s performance with the previous ML approaches.

We used the Python programming language and the SciPy library [25] to implement and validate the classification models based on such algorithms. We applied the random search method to configure the algorithms’ hyperparameters to improve performance carefully. The configurations can be verified in the GitHub repository [12].

### Classification Metrics

We calculated the precision, accuracy score, recall, Brier Score, AUROC, and AUPRC for the classification models [26]. The precision represents the proportion of classifications that are true positives and is given by:


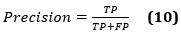


where *TP* is the true positives and *FP* is the false positives. The accuracy score presents fractions of correct classifications and is given by:


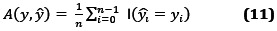


where *A* is the accuracy score, 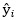 is the classified value of a sample, *y_i_* is the corresponding true value, *n* is the number of samples, and *I*(*x*) is the indicator function.

The recall calculates the actual positives that are correctly positives and is given by:


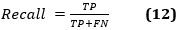


where *FN* is the number of false negatives. It is relevant for evaluating classifications related to diagnosis due to the highly undesired impacts of false negatives.

The Brier Score provides the mean squared difference between predicted probabilities and expected results, given by:


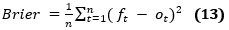


where *f_t_* is the predicted value, *o_t_* is the expected value, and *n* is the number of samples.

Finally, the AUROC provides an overview of the diagnostic abilities of the models. However, the use of the AUPRC is usually recommended when handling problems using unbalanced data.

## Results

The implementations of classification models using the MLP, GBM, DT, RF, XGBoost, KNN, SVM, and LR algorithms are available in the GitHub repository [12]. Using 10-fold cross-validation with five repetitions, the mean values of precision, accuracy score, recall, and Brier Score of the DT-based classification models were among the best results (Table 2). Such models presented similar results using the six data sets. For the *RT-PCR unbalanced/balanced* and *both unbalanced/balanced* data sets, the LR algorithm was outperformed by the other models. In the results, LR and LRR stand for models with weak and strong regularization, respectively.

**Table 2:** Results of 10-fold cross-validation for the classification models using the unbalanced and balanced data sets.

Data sets and models			Precision (%)		Accuracy score (%)		Recall (%)		Brier Score
**RT-PCR^a^ unbalanced and balanced**									
	MLP^b^, unbalanced (balanced)	97.33 (95.86)		96.24 (95.81)		97.08 (95.80)		0.04 (0.04)	
	GBM^c^, unbalanced (balanced)	97.32 (95.95)		96.30 (95.70)		97.17 (95.47)		0.04 (0.04)	
	RF^d^, unbalanced (balanced)	97.42 (96.06)		96.55 (96.00)		97.46 (95.97)		0.04 (0.04)	
	DT^e^, unbalanced (balanced)	97.49 (96.50)		96.33 (95.91)		97.04 (95.32)		0.04 (0.04)	
	XGBoost^f^, unbalanced (balanced)	97.36 (95.94)		96.30 (95.52)		97.13 (95.10)		0.04 (0.04)	
	KNN^g^, unbalanced (balanced)	97.38 (95.92)		96.55 (95.48)		97.50 (95.04)		0.03 (0.05)	
	SVM^h^, unbalanced (balanced)	97.17 (95.84)		96.19 (95.58)		97.18 (95.34)		0.04 (0.04)	
	LRR^i^, unbalanced (balanced)	86.97 (76.86)		86.72 (81.70)		94.37 (90.93)		0.13 (0.18)	
	LR^j^, unbalanced (balanced)	87.00 (76.56)		86.72 (80.63)		94.33 (88.53)		0.13 (0.19)	
**Rapid unbalanced and balanced**									
	MLP, unbalanced (balanced)	99.33 (96.66)		98.70 (95.40)		99.32 (94.10)		0.01 (0.05)	
	GBM, unbalanced (balanced)	99.33 (96.18)		98.72 (95.33)		99.34 (94.50)		0.01 (0.05)	
	RF, unbalanced (balanced)	99.26 (96.42)		98.76 (95.21)		99.44 (93.98)		0.01 (0.05)	
	DT, unbalanced (balanced)	99.37 (95.51)		98.69 (94.59)		99.27 (93.67)		0.01 (0.05)	
	XGBoost, unbalanced (balanced)	99.33 (96.83)		98.72 (95.41)		99.34 (93.94)		0.01 (0.05)	
	KNN, unbalanced (balanced)	99.31 (97.43)		98.84 (94.58)		99.49 (91.63)		0.01 (0.05)	
	SVM, unbalanced (balanced)	99.30 (97.30)		98.73 (95.60)		99.37 (93.85)		0.01 (0.04)	
	LRR, unbalanced (balanced)	96.65 (82.00)		96.23 (84.22)		99.53 (87.93)		0.04 (0.16)	
	LR, unbalanced (balanced)	96.75 (84.75)		96.14 (85.33)		99.32 (86.32)		0.04 (0.15)	
**Both unbalanced and balanced**									
	MLP, unbalanced (balanced)	95.36 (93.53)		94.82 (89.18)		99.20 (84.23)		0.05 (0.11)	
	GBM, unbalanced (balanced)	95.23 (93.67)		94.73 (89.31)		99.25 (84.38)		0.05 (0.11)	
	RF, unbalanced (balanced)	95.31 (93.81)		94.87 (89.22)		99.32 (84.04)		0.05 (0.11)	
	DT, unbalanced (balanced)	95.43 (93.75)		94.79 (89.12)		99.10 (83.87)		0.05 (0.11)	
	XGBoost, unbalanced (balanced)	95.32 (93.60)		94.78 (89.22)		99.21 (84.24)		0.05 (0.11)	
	KNN, unbalanced (balanced)	95.50 (92.77)		91.09 (88.63)		94.81 (83.86)		0.09 (0.11)	
	SVM, unbalanced (balanced)	95.21 (93.36)		94.75 (89.33)		99.30 (84.73)		0.05 (0.11)	
	LRR, unbalanced (balanced)	92.45 (80.79)		92.04 (80.48)		99.48 (80.11)		0.08 (0.20)	
	LR, unbalanced (balanced)	92.49 (82.44)		91.98 (81.08)		99.36 (79.14)		0.08 (0.19)	

^a^RT-PCR: reverse transcription polymerase chain reaction.

^b^MLP: multilayer perceptron.

^c^GBM: gradient boosting machine.

^d^RF: random forest.

^e^DT: decision tree.

^f^XGBoost: extreme gradient boosting.

^g^KNN: k-nearest neighbors.

^h^SVM: support vector machine.

^i^LRR: logistic regression (strong regularization).

^j^LR: logistic regression (weak regularization).

When removing features according to the chi-square results, there was a considerable decrease in the classification models’ performances (Multimedia Appendix 1). However, in general, the classification models continued presenting good performances. For example, the KNN classification model presented the lowest accuracy score (77.42%) using the *RT-PCR balanced* data set. The remaining classification models, considering all data sets, presented accuracy scores between 80.15% and 97.58%. Depending on the preprocessed data set, the LR (weak/strong regularization) continued to be outperformed by the other algorithms. Presenting such scenarios is relevant to analyze how the algorithms behave when models are implemented with reduced reported symptoms.

**Figure 4:**
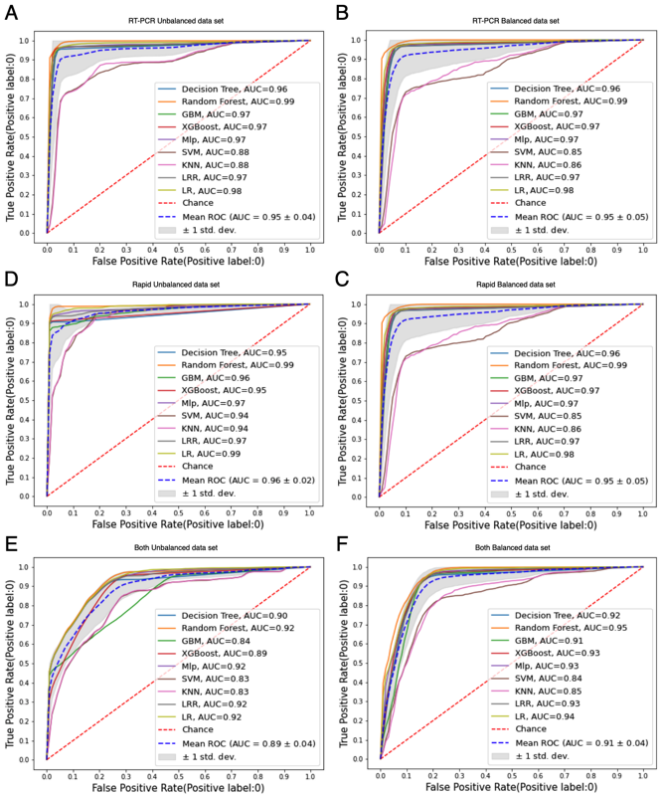
The models' ROC curves with (A) RT-PCR unbalanced, (B) RT-PCR balanced, (C) rapid unbalanced, (D) rapid balanced, (E) both unbalanced, and (F) both balanced. AUC: area under the receiver operating characteristic curve; GBM: gradient boosting machine; KNN: k-nearest neighbors; LR: logistic regression (weak regularization); LRR: logistic regression (strong regularization); Mlp: multilayer perceptron; ROC: receiver operating characteristic; RT-PCR: reverse transcription polymerase chain reaction; SVM: support vector machine; XGBoost: extreme gradient boosting.

In addition, by computing the AUROC using the RT-PCR, rapid, and both test scenarios, the trade-offs between sensitivity (true-positive rate) and probability (false-positive rate) were identified, evidencing the diagnostic abilities of the classification models when the discrimination threshold is varied (Figure 4). The classification models presented high discriminatory power for all scenarios, with the curves closer to each graphic representation’s upper left corner. However, for such scenarios, the KNN and SVM classification models presented the lowest discriminatory power.

**Figure 5:**
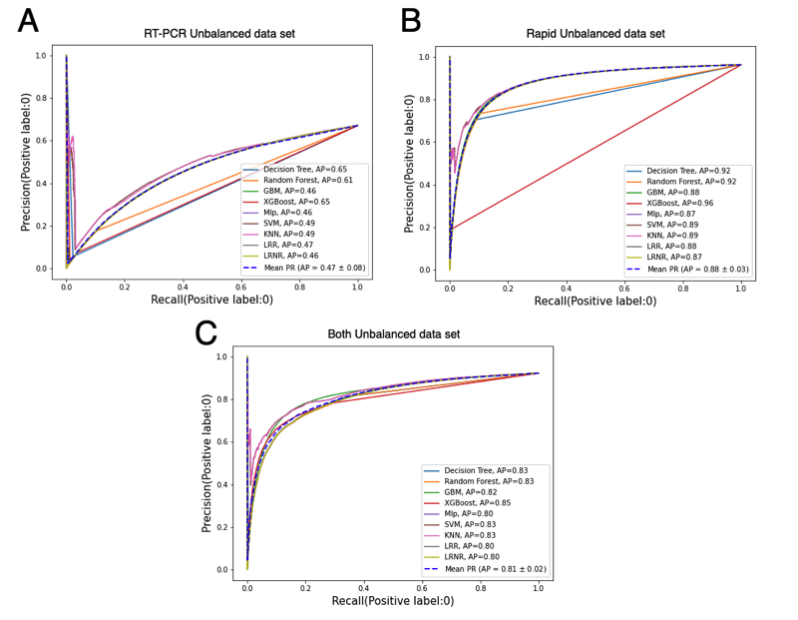
Models' precision-recall curve with (A) RT-PCR unbalanced data set, (B) rapid unbalanced data set, and (C) both unbalanced data set. AP: average precision; GBM: gradient boosting machine; KNN: k-nearest neighbors; LR: logistic regression (weak regularization); LRR: logistic regression (strong regularization); Mlp: multilayer perceptron; PR: precision-recall; RT-PCR: reverse transcription polymerase chain reaction; SVM: support vector machine; XGBoost: extreme gradient boosting.

Given the three unbalanced data sets, there were more negative than positive COVID-19 cases. We computed the AUPRC to verify the classification models when handling the minority class, analyzing the trade-off between precision and recall for different decision thresholds (Figure 5). The AUPRC was summarized using the average precision (AP), as a weighted mean of precision. The *RT-PCR unbalanced* data set was mildly unbalanced, with a baseline AUPRC of 0.33. The *rapid unbalanced* data set was highly unbalanced, with a baseline AUPRC of 0.04. This was also the case for the *both unbalanced* data set, with a baseline AUPRC of 0.08. The DT and XGBoost achieved the best AP value (65%) using the *RT-PCR unbalanced* data set. For the remaining scenarios, the classification models presented AP values between 80% and 96%.

**Figure 6:**
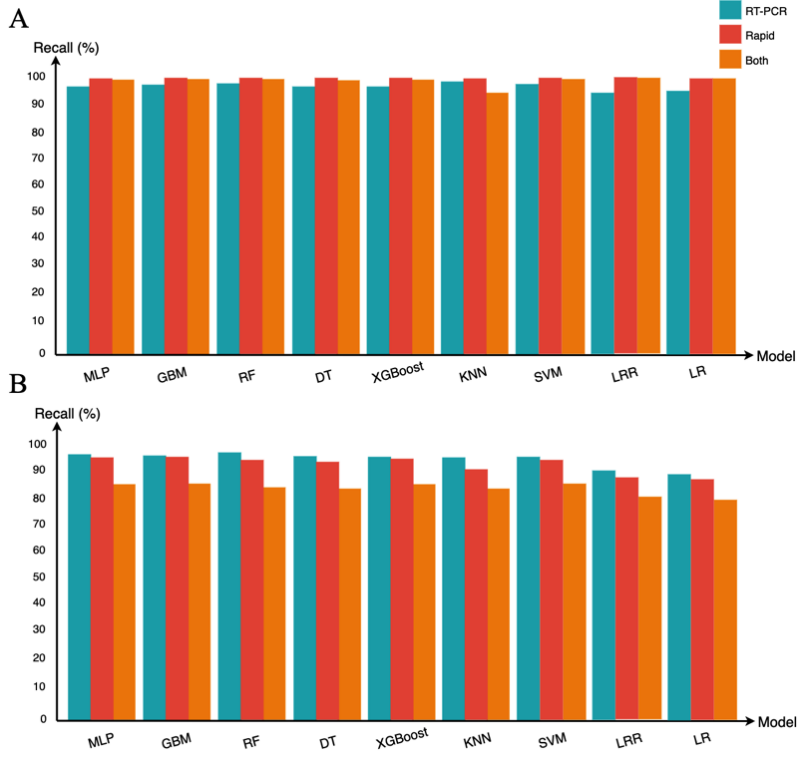
(A) The mean recall for the MLP, GBM, RF, DT, XGBoost, KNN, SVM, LRR, and LR classification models using the unbalanced data sets for RT-PCR, rapid, and both types. (B) The mean recall for the MLP, GBM, RF, DT, XGBoost, KNN, SVM, LRR, and LR classification models using the balanced data sets for RT-PCR, rapid, and both types. DT: decision tree; GBM: gradient boosting machine; KNN: k-nearest neighbors; LR: logistic regression (weak regularization); LRR: logistic regression (strong regularization); MLP: multilayer perceptron; RF: random forest; RT-PCR: reverse transcription polymerase chain reaction; SVM: support vector machine; XGBoost: extreme gradient boosting.

We also applied the Friedman and Nemenyi tests to improve confidence in evaluating the classification models, observing that the experiments’ results were statistically significant. The classification models were compared over the 6 data sets using the Friedman test [27]. This comparison focused on the recall results due to the highly undesired impacts of false negatives in the COVID-19 application scenario (Figure 6). The null hypothesis was that all classification models are equivalent and have equal mean ranks. The tests resulted in a *P*<.001 for the *RT-PCR unbalanced* (*t*=307.16), *RT-PCR balanced* (*t*=328.72), *rapid unbalanced* (*t*=247.43), *rapid balanced* (*t*=239.20), *both unbalanced* (*t*=226.98), and *both balanced* (*t*=343.10) data sets. The results showed that the difference between the mean recall values was probably real (*P*≤.1). The Friedman test ranked the classification models for each data set, resulting in an average rank for each classification model.

Based on the Friedman test results, the Nemenyi test [27] was applied to compare the classification models using the mean ranks. The critical difference (CD) between the classification models was verified using the Nemenyi test, with *α*=0.1. The CD is relevant to highlight if the classification models are separated by an interval less than the CD, meaning that the classification models were statistically indistinguishable. Thus, for most of the data sets, the difference between LRR/LR (statistically indistinguishable) and the other classification models was highlighted by the CD using the mean recall results (Multimedia Appendix 2). Depending on the data set, MLP and GBM were also statistically indistinguishable, as was the case of DT, RF, XGBoost, KNN, and SVM.

From the classification metrics results and the Friedman and Nemenyi tests (Figure 6), the top five features of the classification models with the highest performances (ie, MLP, GBM, DT, RF, XGBoost, and SVM) were ranked using the permutation feature importance method. Each average importance and SD values were presented for the DT-based classification models and the RT-PCR, rapid, and both types scenarios (Table 3). The average importance and SD information relate to reducing the feature importance when a feature is not considered. For example, according to the frequency of symptoms and the number of confirmed cases (Figure 3), *fever* showed higher average importance values for almost all scenarios than other reported symptoms. We also applied the permutation feature importance method for the unbalanced data sets (Multimedia Appendix 3).

We also present the results achieved using the permutation feature importance method for detailing the feature ranking for classifications with MLP and SVM models (Table 4). For example, similar to the DT-based classification models, *fever* presented higher average importance values for almost all test scenarios than other symptoms reported by patients. For such algorithms, we also present the average importance and SD for the unbalanced data sets (Multimedia Appendix 3).

Therefore, the top five most significant features vary depending on the algorithm used to implement the classification model (Table 5). For the *RT-PCR balanced* data set, all algorithms prioritized the same top two features (ie, *fever* and *gender*), slightly differing in the top three and top five, while, for the *rapid balanced* data set, all algorithms prioritized the same top two features (ie, *dyspnea* and *olfactory disorders*), also slightly different in the top three, top four, and top five. For the *both balanced* data set, the algorithms prioritized the top two features similar to the classifications with the *RT-PCR balanced* data set. We also applied the permutation feature importance method to rank features using the unbalanced data sets (Multimedia Appendix 3).

In addition, to improve the experiments conducted to assist the COVID-19 test prioritization, we combined the classification models to define voting ensemble models using the majority voting strategy (Multimedia Appendix 4). Two combinations of classification models were considered for each data set: DT-based models (ie, GBM, DT, RF, and XGBoost) and non-DT models (ie, MLP, SVM, KNN, LRR, and LR). In general, for the voting ensemble models implemented with the six data sets, the mean results of classification metrics using 10-fold cross-validation were similar to those of the MLP, GBM, DT, RF, XGBoost, KNN, SVM, LRR, and LR models (Table 2).

**Table 3:** The average importance and SD values for each feature for the decision tree–based classification models using the balanced data sets.

Data sets and features		GBM^a^, mean (SD)	DT^b^, mean (SD)	RF^c^, mean (SD)	XGBoost^d^, mean (SD)
**RT-PCR^e^ balanced**					
	Gender	0.233 (0.013)	0.245 (0.013)	0.238 (0.013)	0.233 (0.013)
	Health professional	0.045 (0.005)	0.055 (0.005)	0.051 (0.005)	0.042 (0.005)
	Fever	0.260 (0.012)	0.263 (0.012)	0.267 (0.013)	0.262 (0.013)
	Sore throat	0.100 (0.007)	0.102 (0.007)	0.100 (0.007)	0.101 (0.006)
	Dyspnea	0.096 (0.007)	0.100 (0.007)	0.098 (0.007)	0.092 (0.007)
	Olfactory disorders	0.026 (0.004)	0.029 (0.003)	0.014 (0.004)	0.016 (0.003)
	Cough	0.087 (0.007)	0.096 (0.007)	0.084 (0.007)	0.082 (0.008)
	Coryza	0.026 (0.004)	0.027 (0.004)	0.022 (0.004)	0.014 (0.003)
	Taste disorders	0.030 (0.004)	0.040 (0.004)	0.027 (0.004)	0.024 (0.003)
	Headache	0.021 (0.004)	0.024 (0.005)	0.018 (0.004)	0.002 (0.003)
**Rapid balanced**					
	Gender	0.135 (0.009)	0.109 (0.009)	0.122 (0.010)	0.123 (0.010)
	Health professional	0.026 (0.005)	0.027 (0.005)	0.020 (0.004)	0.027 (0.005)
	Fever	0.120 (0.012)	0.124 (0.012)	0.114 (0.011)	0.109 (0.010)
	Sore throat	0.019 (0.005)	0.030 (0.005)	0.023 (0.004)	0.013 (0.004)
	Dyspnea	0.184 (0.012)	0.179 (0.012)	0.187 (0.013)	0.179 (0.013)
	Olfactory disorders	0.175 (0.012)	0.178 (0.013)	0.180 (0.014)	0.154 (0.012)
	Cough	0.076 (0.009)	0.084 (0.011)	0.080 (0.008)	0.080 (0.008)
	Coryza	0.090 (0.008)	0.092 (0.009)	0.087 (0.007)	0.052 (0.005)
	Taste disorders	0.078 (0.008)	0.081 (0.009)	0.053 (0.007)	0.071 (0.007)
	Headache	0.035 (0.007)	0.030 (0.007)	0.035 (0.006)	0.035 (0.007)
**Both balanced**					
	Gender	0.159 (0.007)	0.160 (0.007)	0.153 (0.007)	0.156 (0.007)
	Health professional	0.025 (0.004)	0.024 (0.004)	0.023 (0.004)	0.025 (0.004)
	Fever	0.211 (0.010)	0.215 (0.011)	0.213 (0.010)	0.209 (0.010)
	Sore throat	0.080 (0.005)	0.081 (0.005)	0.082 (0.005)	0.078 (0.005)
	Dyspnea	0.077 (0.006)	0.075 (0.005)	0.073 (0.005)	0.076 (0.005)
	Olfactory disorders	0.059 (0.006)	0.050 (0.005)	0.050 (0.005)	0.046 (0.005)
	Cough	0.060 (0.005)	0.058 (0.005)	0.054 (0.005)	0.060 (0.005)
	Coryza	0.047 (0.004)	0.042 (0.003)	0.040 (0.003)	0.044 (0.004)
	Taste disorders	0.063 (0.006)	0.079 (0.006)	0.072 (0.005)	0.069 (0.005)
	Headache	0.042 (0.005)	0.046 (0.005)	0.044 (0.005)	0.045 (0.005)

^a^GBM: gradient boosting machine.

^b^RF: random forest.

^c^DT: decision tree.

^d^XGBoost: extreme gradient boosting.

^e^RT-PCR: reverse transcription polymerase chain reaction.

**Table 4:** The average importance and SD for each feature for the MLP and SVM models and the balanced data sets.

Data sets and features		MLP^a^, mean (SD)		SVM^b^, mean (SD)
**RT-PCR^c^ balanced**				
	Gender	0.236 (0.013)	0.230 (0.013)	
	Health professional	0.048 (0.005)	0.042 (0.005)	
	Fever	0.262 (0.013)	0.257 (0.013)	
	Sore throat	0.103 (0.006)	0.098 (0.006)	
	Dyspnea	0.096 (0.007)	0.088 (0.007)	
	Olfactory disorders	0.027 (0.004)	0.015 (0.004)	
	Cough	0.084 (0.008)	0.078 (0.007)	
	Coryza	0.025 (0.004)	0.013 (0.004)	
	Taste disorders	0.031 (0.004)	0.020 (0.004)	
	Headache	0.023 (0.003)	0.002 (0.003)	
**Rapid balanced**				
	Gender	0.120 (0.009)	0.117 (0.010)	
	Health professional	0.033 (0.006)	0.029 (0.005)	
	Fever	0.115 (0.010)	0.105 (0.011)	
	Sore throat	0.012 (0.005)	0.023 (0.004)	
	Dyspnea	0.177 (0.013)	0.177 (0.014)	
	Olfactory disorders	0.157 (0.012)	0.149 (0.012)	
	Cough	0.082 (0.009)	0.076 (0.008)	
	Coryza	0.064 (0.006)	0.058 (0.006)	
	Taste disorders	0.072 (0.007)	0.055 (0.006)	
	Headache	0.036 (0.006)	0.028 (0.005)	
**Both balanced**				
	Gender	0.161 (0.007)	0.154 (0.007)	
	Health professional	0.025 (0.004)	0.024 (0.004)	
	Fever	0.207 (0.010)	0.193 (0.009)	
	Sore throat	0.084 (0.005)	0.075 (0.005)	
	Dyspnea	0.078 (0.006)	0.088 (0.006)	
	Olfactory disorders	0.055 (0.006)	0.049 (0.005)	
	Cough	0.062 (0.005)	0.071 (0.005)	
	Coryza	0.035 (0.003)	0.046 (0.003)	
	Taste disorders	0.071 (0.006)	0.068 (0.005)	
	Headache	0.051 (0.005)	0.045 (0.005)	

^a^MLP: multilayer perceptron.

^b^SVM: support vector machine.

^c^RT-PCR: reverse transcription polymerase chain reaction.

**Table 5:** The five most significant factors for COVID-19 test prioritization using the classification models with the highest performances and the data sets.

Data sets and models		Top one	Top two	Top three	Top four	Top five
**RT-PCR^a^ balanced**						
	MLP^b^	Fever	Gender	Sore throat	Dyspnea	Cough
	GBM^c^	Fever	Gender	Sore throat	Dyspnea	Cough
	RF^d^	Fever	Gender	Sore throat	Dyspnea	Cough
	DT^e^	Fever	Gender	Sore throat	Dyspnea	Cough
	XGBoost^f^	Fever	Gender	Sore throat	Dyspnea	Cough
	SVM^g^	Fever	Gender	Sore throat	Dyspnea	Cough
**Rapid balanced**						
	MLP	Dyspnea	Olfactory disorders	Gender	Fever	Cough
	GBM	Dyspnea	Olfactory disorders	Gender	Fever	Coryza
	RF	Dyspnea	Olfactory disorders	Gender	Fever	Coryza
	DT	Dyspnea	Olfactory disorders	Fever	Gender	Coryza
	XGBoost	Dyspnea	Olfactory disorders	Gender	Fever	Cough
	SVM	Dyspnea	Olfactory disorders	Gender	Fever	Cough
**Both balanced**						
	MLP	Fever	Gender	Sore throat	Dyspnea	Taste disorders
	GBM	Fever	Gender	Sore throat	Dyspnea	Taste disorders
	RF	Fever	Gender	Sore throat	Dyspnea	Taste disorders
	DT	Fever	Gender	Sore throat	Taste disorders	Dyspnea
	XGBoost	Fever	Gender	Sore throat	Dyspnea	Taste disorders
	SVM	Fever	Gender	Dyspnea	Sore throat	Cough

^a^RT-PCR: reverse transcription polymerase chain reaction.

^b^MLP: multilayer perceptron.

^c^GBM: gradient boosting machine.

^d^RF: random forest.

^e^DT: decision tree.

^f^XGBoost: extreme gradient boosting.

^g^SVM: support vector machine.

## Discussion

### Principal Findings

The raw data set’s data preprocessing enabled the implementation, validation, and comparison of classification models with different characteristics such as using neural layers, tree combinations, and calculating the distance between data. The preprocessing also resulted in the public data availability of patients who were symptomatic tested using RT-PCR and rapid tests [10]. Thus, the data sets can be reused by other studies to improve the state of the art.

The algorithms were trained and tested using the unbalanced and balanced data sets, improving data representativity. The best classification metrics results were related to the RT-PCR and rapid tests scenarios using unbalanced and balanced data. Although the classification models’ performance was similar for the RT-PCR and rapid tests scenarios, the RT-PCR test scenario is the most clinically relevant one due to the RT-PCR testing’s high confidence. The RT-PCR test’s precision increases confidence in the diagnosis, even if the patient was tested in the first days after symptoms onset. For both test scenarios with unbalanced data, although presenting a low Brier Score and high precision, accuracy score, and recall, the classification models presented a lower AUROC because of the higher negative than positive COVID-19 cases. For both test scenarios with balanced data, the Brier score continued to be low. The precision, accuracy, and AUROC were higher; however, the recall results were slightly decreased if compared to the unbalanced data results.

The recall metric is relevant due to the undesired impacts of false negatives in clinical practice. Thus, we improved the classification models’ quality of comparisons by applying the Friedman and Nemenyi tests based on the six data sets’ recall. We used such statistical comparison results for defining the MLP, GBM, DT, RF, XGBoost, and SVM as the classification models with the highest performances for COVID-19 test prioritization in Brazil.

Given the classification models with the highest performances and the five most significant features for COVID-19 test prioritization, the fever’s importance as one of the top two features is according to the aforementioned statistics (Figure 3). The statistics showed that fever was the second most frequent symptom reported by patients who were symptomatic, confirmed as COVID-19 cases. *Gender* and *dyspnea* were also among the highest-ranked features used by classification models. For example, for the *RT-PCR balanced* data set, observing the DT model’s decision rules to get an overview of the role of gender in classifications, positive or negative decisions for males and females differed based on reported symptoms and the *health professional* feature. However, further investigation about the role of gender in classifications is recommended for future works.

Therefore, secondary RQ 1 was answered by showing that *gender* and *health professional* features are related to relevant demographic characteristics to support the COVID-19 test prioritization in Brazil (Tables 4 and 5). Secondary RQ 2 was also answered, showing that fever, sore throat, dyspnea, olfactory disorders, cough, coryza, taste disorders, and headache are relevant symptoms.

All DT-based classification models considered in this study are among the classification models with the highest performances, grouped based on the results of classification metrics and statistical tests. This fact is relevant due to the high levels of DTs’ interpretability, positively impacting health care professionals’ final decision making. In clinical practice, ML-based applications’ acceptance increases when health care professionals can easily understand and interpret classification models’ outputs to track decision-making logic [28]. Given the grouping of models with similar performances, we used the criterion of easy interpretability to answer secondary RQ 3. Thus, the DT classification model was considered the most suitable for COVID-19 test prioritization in Brazil. We configured the model with the Gini impurity criterion, best split strategy, no maximum depth, a minimum number of two samples split and one sample leaf, no minimum weighted fraction leaves and no impurity decrease and split, unlimited number of features and leaves, global random state instance, no class weight, and no pruning. As one of the classification models with the highest performances, DT provides a simple tree representation of the decision making, enabling a unique tree’s straightforward interpretation by health care professionals.

To answer secondary RQ 4, we analyzed the DT model’s classification results, observing that a considerable fraction of the incorrectly classified instances occurred when patients reported only one, two, or three symptoms. Furthermore, we conducted an experiment to verify the impacts of reducing features in the performance of the implemented classification models (Multimedia Appendix 1). For example, with the *both RT-PCR balanced* data set, when the symptoms of sore throat, dyspnea, headache, and coryza were not considered to implement the DT classification model, the performance results decreased considerably. This reduces the ability of the model to distinguish between positive and negative cases.

Although the DT is considered the most suitable model, all the other classification models that presented high performance were relevant to address COVID-19 test prioritization. In Brazil, due to other epidemics (eg, dengue fever [29]), many people report symptoms that may or may not be related to COVID-19. As a limited-income country, Brazil also has inefficient testing strategies such as shortages of COVID-19 tests. One of the available classification models can be applied for COVID-19 test prioritization during primary health care, with a mean accuracy score of at least 88.63%.

### Comparison With Prior Work

The relevance of research addressing viral infection outbreaks is evidenced from the public administration (eg, surveillance systems) to the diagnosis viewpoint. For example, Son et al [30] used a South Korean time series of influenza incidence for early outbreak detection, aiming to assist the definition of control policies. Chatterjee et al [31] analyzed COVID-19 data sets to identify risks of spreading, identify correlated factors associated with the disease’s spread, identify the impact of social isolation, and experiment with univariate long short-term memory models for forecasting of total cases and total deaths. In general, infectious disease research is guided by trends in data analytics [32].

Indeed, the COVID-19 pandemic is an example of a problematic scenario. Kumar [33] applied cluster analysis to study and improve the monitoring of SARS-CoV-2 infections in India, providing insights on clusters of affected Indian states and union territories. Besides aiming to improve the management of available resources, Khakharia et al [34] developed outbreak classification models for COVID-19 using data sets with information about patients who live in India, Bangladesh, the Democratic Republic of Congo, Pakistan, China, Philippines, Germany, Indonesia, Ethiopia, and Nigeria. Vaid et al [35] implemented and validated models (eg, XGBoost) to predict mortality and critical events using electronic health records of patients who tested positive for COVID-19 in New York City.

To assist COVID-19 detection, Brinati et al [36] validated models implemented using DT, extremely randomized trees, KNN, LR, naive Bayes, RF, and three-way RF algorithms. The authors considered COVID-19 detection using routine blood exams, gender, and age. The accuracy of the models ranged between 82% and 86%. However, the large number of required blood exams (ie, 13) was a limitation, which may compromise this approach’s feasibility in low- and middle–income countries.

Ahamad et al [21] used a Chinese data set to assist the COVID-19 detection considering symptoms (ie, fever, cough, pneumonia, lung infection, coryza, muscle soreness, and diarrhea), gender, age, travel history, and isolation. The authors validated the XGBoost, SVM, DT, RF, and GBM models. XGBoost presented the highest accuracy with more than 85%, varying according to age. However, lung infection use, detected by chest images, increases costs and may limit the disease’s rapid screening.

Aiming to improve confidence in screening COVID-19, Mei et al [37] used computerized tomography (CT) images along with symptoms (ie, fever, cough, and cough with sputum), exposure history, laboratory testing (ie, white blood cells, neutrophils, percentage neutrophils, lymphocytes, and percentage lymphocytes), age, gender, and temperature. They applied the deep convolutional neural network to analyze images, besides comparing the performance of SVM, RF, and MLP models, showing that MLP presented the highest accuracy score. Afterward, the authors combined images and clinical information. Similarly, requiring images increases costs and may limit the rapid screening of COVID-19 in low- and middle–income countries.

Finally, Zoabi et al [4] used gender, age, symptoms (eg, cough, fever, sore throat, shortness of breath, and headache), and contact with a confirmed case to classify positive and negative COVID-19 cases. The authors implemented a GBM model based on data reported by the Israeli Ministry of Health. The GBM model presented an AUROC of 86% and 90% using a reduced set of features and the complete set, respectively. Similar to our study, the authors reported the high importance of gender during the classifications. We also improved the state of the art by presenting a comparison of other implementations of classification models. Besides cough, fever, sore throat, shortness of breath, and headache, we used the symptoms of olfactory disorders, coryza, and taste disorders to improve the results.

In contrast to such prior works, we focused on raw data from 55,676 Brazilians and used features that do not require expensive exams such as CT images and blood tests. Symptoms included fever, sore throat, dyspnea, olfactory disorders, cough, coryza, taste disorders, and headache. The *gender* and *health professional* features were the additional information required to conduct the COVID-19 test prioritization using the classification models. *Gender* was also used as a feature by prior works [4,20,36,37]. The use of exams such as CT images and blood tests limits classification models’ application scenarios because it is necessary to prioritize patients who are symptomatic for testing in the first days after symptoms onset.

### Limitations

By preprocessing the 55,676 raw data, the *RT-PCR balanced* data set only included 1832 patients who were symptomatic, the *rapid balanced* data set included 1296 patients who were symptomatic, and the *both balanced* data set included 3128 patients who were symptomatic. However, to improve the strength of results and decrease size limitation, we also considered 3 unbalanced data sets. For example, the *both unbalanced* data set was composed of 20,021 patients who were symptomatic and tested for COVID-19.

Furthermore, in a real-world scenario, the number of patients who were asymptomatic with COVID-19 can also be considered a limitation to the classification models’ applicability. In this case, this study continues to be relevant due to the remaining symptomatic cases that also require health care professionals and the government’s attention. The evaluation of patients who are symptomatic is also relevant to prevent the unplanned use of COVID-19 testing resources due to other disease outbreaks in Brazil caused by other viral infections (eg, dengue, Zika, and chikungunya). Such viral infections present similar symptoms that may complicate health care professionals’ decision on the adequate testing type needed.

The reduced number of symptoms reported by a patient who is symptomatic can also negatively impact the reuse classification models. Nevertheless, the feature ranking and other information (eg, contact with infected people) are relevant to complement the classification models during the decision making conducted by health care professionals and policy makers. We verified the impacts of reducing features in the performance of implemented classification models (Multimedia Appendix 1).

Finally, the number of classification models implemented, validated, and compared is another limitation of our study, given the wide variety of available algorithms and ensemble strategies. This limitation was reduced by selecting well-known algorithms based on trees, linear regression, statistical learning, distance, and the concept of neurons.

### Clinical Practice Context

The availability of eHealth and mHealth systems is relevant to assist decision making in different scenarios. One such scenario is detecting COVID-19 in patients who reside in remote and hard-to-reach locations (eg, Amazonia or Latin America) [38]. Developers can integrate eHealth and mHealth systems with services that enable health care professionals to be alerted when the risk of disease is detected. The use of eHealth and mHealth systems should be encouraged, considering that the early detection of COVID-19 is essential in clinical practice to enable early medical attention, possibly reducing the negative impacts of late treatments. This type of eHealth and mHealth system can also benefit public health systems when factors related to the human condition (eg, fatigue and lack of experience) and the collapse of health services negatively influence health care professionals’ decision making during patients’ evaluation. Such scenarios are authentic in the context of the COVID-19 pandemic [39].

Therefore, the implemented classification models can be the basis for eHealth and mHealth systems to support health care professionals and policy makers during the COVID-19 test prioritization. To be applied in clinical practice and integrated with the current clinical workflow, the availability of the DT classification model and the use of feature ranking information through web services to be consumed by an eHealth or mHealth system is recommended. Such a system shall present classification results for health care professionals in a user-friendly manner. The straightforward interpretation of classification models is relevant to increase health care professionals’ confidence in classification results. For example, the web services can be integrated with Brazilian public health facilities’ systems to prioritize the reduced COVID-19 testing resources.

**Figure 7:**
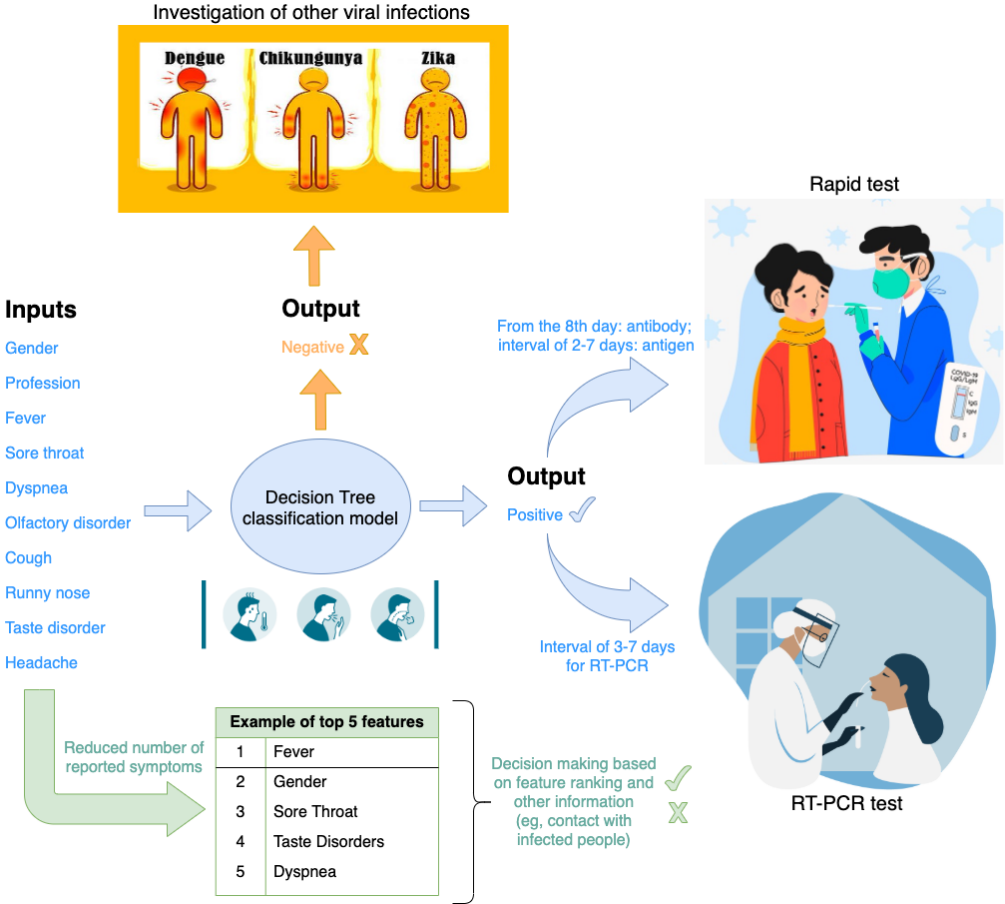
An application scenario to connect the decision tree classification model with a clinical workflow. The model guides the test prioritization of patients who were symptomatic suspected of COVID-19. RT-PCR: reverse transcription polymerase chain reaction.

We present an application scenario integrating a clinical workflow and the DT classification model (Figure 7). The DT is used to prioritize patients who are symptomatic for COVID-19 testing. However, when the number of reported symptoms is too low, the classification models cannot distinguish between positive and negative cases. In this case, health care professionals can reuse the feature ranking and other information (eg, contact with infected people) to make decisions about COVID-19 testing. Thus, the use of feature ranking information is guided by the answer of secondary RQ 4. If the result is not prioritized, the patient’s clinical condition should be further investigated in regard to other viral diseases.

For the application scenario, there are five possible flows: (1) confirmed case with classification model and rapid test result, (2) confirmed case with classification model and RT-PCR test result, (3) confirmed case using feature ranking and rapid test result, (4) confirmed case using feature ranking and RT-PCR test result, and (5) negative case with the recommendation of investigation of other viral diseases. It is relevant to consider the days between the onset of symptoms and COVID-19 testing: closed interval of 3-7 days for RT-PCR test, from the eighth day for the rapid antibody test, and closed interval of 2-7 days for the rapid antigen test [40-42].

### Conclusions

The results showed the relevance of using classification models for COVID-19 test prioritization in Brazil, mainly based on the symptoms that do not require expensive exams. By comparing the classification models using raw data from 55,676 Brazilians, the 10-fold cross-validation method, classification metrics, and the Friedman and Nemenyi tests, the MLP, GBM, DT, RF, XGBoost, and SVM presented the highest performances with similar results.

DT-based classification models’ high performances are relevant for our application scenario due to the high levels of DTs’ interpretability, positively impacting health care professionals’ final decision making. Therefore, applying the easy interpretability as an additional comparison criterion, DT was considered the most suitable classification model, effectively assisting in the decision making for prioritizing patients who are symptomatic for testing. Information about the features *gender*, *health professional*, *fever*, *sore throat*, *dyspnea*, *olfactory disorders*, *cough*, *coryza*, *taste disorders*, and *headache* enable the COVID-19 test prioritization for patients who are symptomatic. The use of symptoms that do not require expensive exams contributes to assisting patients who live, for example, in needy and hard-to-reach communities. The results of feature ranking reported in this paper are also relevant to support a more detailed analysis in a scenario where a patient reports a reduced number of symptoms.

To improve testing prioritization, we plan to investigate the relationship between the symptoms reported by patients with COVID-19 and other widespread diseases in Brazil, such as dengue fever, Zika fever, and chikungunya. Thus, we aim to include implementing and validating classification models and developing and validating an eHealth system to support health care professionals and policy makers in decision making for testing strategies.

## Appendix

Multimedia Appendix 1 Performance of classification models considering the chi-square results.

Multimedia Appendix 2 Results of statistical tests.

Multimedia Appendix 3 Feature ranking results for the unbalanced data set.

Multimedia Appendix 4 Mean values of classification metrics for the ensemble models.

## Abbreviations

AP: average precision
AUPRC: area under the precision-recall curve
AUROC: area under the receiver operating characteristic curve
CD: critical difference
CT: computerized tomography
DT: decision tree
GBM: gradient boosting machine
KNN: k-nearest neighbors
LR: logistic regression
LRR: logistic regression (strong regularization)
mHealth: mobile health
ML: machine learning
MLP: multilayer perceptron
RF: random forest
RQ: research question
RT-PCR: reverse transcriptase polymerase chain reaction
SVM: support vector machine
XGBoost: extreme gradient boosting
