# Supplementary material for: Classification Models for COVID-19 Test Prioritization in Brazil: Machine Learning Approach
Source: J Med Internet Res. 2021 Apr 8;23(4):e27293. doi: 10.2196/27293 (PMC8034680; doi:10.2196/27293)
Supplement: Multimedia Appendix 1 [file jmir_v23i4e27293_app1.docx]

SUPPLEMENTARY TABLES

Table S1. Results of 10-fold cross validation, removing headache for the classification models using the *Both Unbalanced* dataset.

| Datasets and Models | Precision % | Accuracy Score % | Recall % | AUROC % | Brier Score |
| --- | --- | --- | --- | --- | --- |
| MLP, % and % | 94.74 | 93.84 | 98.81 | 67.03 | 0.06 |
| GBM, % and % | 94.67 | 93.81 | 98.84 | 66.62 | 0.06 |
| RF, % and % | 94.71 | 93.89 | 98.89 | 66.85 | 0.06 |
| DT, % and % | 94.85 | 93.84 | 98.67 | 67.76 | 0.06 |
| XGBoost, % and % | 94.71 | 93.83 | 98.83 | 66.84 | 0.06 |
| KNN, % and % | 95.36 | 88.56 | 92.08 | 69.54 | 0.11 |
| SVM, % and % | 92.46 | 92.01 | 99.44 | 51.92 | 0.07 |
| LRR, % and % | 92.49 | 91.97 | 99.34 | 52.12 | 0.08 |
| LR, % and % | 92.27 | 91.54 | 99.13 | 50.59 | 0.08 |

Table S2. Results of 10-fold cross validation, removing headache, for the classification models using the *Both Balanced* dataset.

| Datasets and Models | Precision % | Accuracy Score % | Recall % | AUROC % | Brier Score |
| --- | --- | --- | --- | --- | --- |
| MLP, % and % | 91.41 | 87.68 | 83.25 | 87.69 | 0.12 |
| GBM, % and % | 91.48 | 87.84 | 83.51 | 87.85 | 0.12 |
| RF, % and % | 91.57 | 87.80 | 83.34 | 87.85 | 0.12 |
| DT, % and % | 91.70 | 87.62 | 82.78 | 87.62 | 0.12 |
| XGBoost, % and % | 91.57 | 87.80 | 83.34 | 87.81 | 0.12 |
| KNN, % and % | 90.74 | 96.72 | 81.88 | 86.72 | 0.13 |
| SVM, % and % | 91.40 | 87.63 | 83.14 | 87.84 | 0.12 |
| LRR, % and % | 80.73 | 80.43 | 80.08 | 80.44 | 0.20 |
| LR, % and % | 82.23 | 81.00 | 79.23 | 81.01 | 0.19 |

Table S3. Results of 10-fold cross validation, removing sore throat, dyspnea, headache, and coryza, for the classification models using the *RT-PCR Unbalanced* dataset.

| Datasets and Models | Precision % | Accuracy Score % | Recall % | AUROC % | Brier Score |
| --- | --- | --- | --- | --- | --- |
| MLP, % and % | 87.87 | 88.03 | 95.35 | 84.26 | 0.12 |
| GBM, % and % | 87.88 | 87.99 | 95.26 | 84.24 | 0.12 |
| RF, % and % | 87.80 | 87.98 | 95.36 | 84.16 | 0.12 |
| DT, % and % | 87.87 | 87.78 | 94.91 | 84.10 | 0.12 |
| XGBoost, % and % | 87.95 | 88.06 | 95.27 | 84.33 | 0.12 |
| KNN, % and % | 87.14 | 86.90 | 94.60 | 82.92 | 0.13 |
| SVM, % and % | 87.75 | 87.95 | 95.39 | 84.12 | 0.12 |
| LRR, % and % | 87.03 | 86.77 | 94.36 | 82.85 | 0.13 |
| LR, % and % | 87.08 | 86.72 | 94.20 | 82.86 | 0.13 |

Table S4. Results of 10-fold cross validation, removing dyspnea, cough, headache, and coryza, for the classification models using the *RT-PCR Balanced* dataset.

| Datasets and Models | Precision % | Accuracy Score % | Recall % | AUROC % | Brier Score |
| --- | --- | --- | --- | --- | --- |
| MLP, % and % | 82.07 | 84.57 | 88.68 | 84.57 | 0.15 |
| GBM, % and % | 81.96 | 84.48 | 88.66 | 84.46 | 0.16 |
| RF, % and % | 88.02 | 84.54 | 88.68 | 84.47 | 0.16 |
| DT, % and % | 82.27 | 84.66 | 88.57 | 84.67 | 0.15 |
| XGBoost, % and % | 81.99 | 84.48 | 88.64 | 84.48 | 0.16 |
| KNN, % and % | 91.36 | 77.42 | 60.96 | 77.43 | 0.23 |
| SVM, % and % | 82.07 | 84.53 | 88.59 | 84.52 | 0.15 |
| LRR, % and % | 76.81 | 81.80 | 91.33 | 81.80 | 0.18 |
| LR, % and % | 76.24 | 80.15 | 87.88 | 80.15 | 0.20 |

Table S5. Results of 10-fold cross validation, removing sore throat and health professional, for the classification models using the *Rapid Unbalanced* dataset.

| Datasets and Models | Precision % | Accuracy Score % | Recall % | AUROC % | Brier Score |
| --- | --- | --- | --- | --- | --- |
| MLP, % and % | 98.92 | 97.53 | 98.51 | 85.50 | 0.02 |
| GBM, % and % | 98.92 | 97.55 | 98.52 | 85.54 | 0.02 |
| RF, % and % | 98.93 | 97.56 | 98.52 | 85.70 | 0.02 |
| DT, % and % | 99.05 | 97.58 | 98.43 | 87.14 | 0.02 |
| XGBoost, % and % | 98.92 | 97.54 | 98.51 | 85.55 | 0.02 |
| KNN, % and % | 98.32 | 97.32 | 98.91 | 77.85 | 0.03 |
| SVM, % and % | 98.91 | 97.56 | 98.55 | 85.41 | 0.02 |
| LRR, % and % | 96.64 | 96.22 | 99.53 | 55.52 | 0.04 |
| LR, % and % | 96.75 | 96.16 | 99.33 | 57.05 | 0.04 |

Table S6. Results of 10-fold cross validation, removing sore throat, for the classification models using the *Rapid Balanced* dataset.

| Datasets and Models | Precision % | Accuracy Score % | Recall % | AUROC % | Brier Score |
| --- | --- | --- | --- | --- | --- |
| MLP, % and % | 96.01 | 94.58 | 93.02 | 94.58 | 0.05 |
| GBM, % and % | 95.54 | 94.53 | 93.51 | 94.55 | 0.05 |
| RF, % and % | 95.91 | 94.41 | 92.87 | 94.40 | 0.06 |
| DT, % and % | 95.71 | 93.72 | 91.60 | 93.75 | 0.06 |
| XGBoost, % and % | 95.99 | 94.50 | 92.96 | 94.51 | 0.05 |
| KNN, % and % | 97.29 | 94.50 | 91.60 | 94.51 | 0.05 |
| SVM, % and % | 95.77 | 94.41 | 93.02 | 94.41 | 0.06 |
| LRR, % and % | 81.01 | 83.67 | 88.17 | 83.67 | 0.16 |
| LR, % and % | 84.66 | 85.33 | 86.48 | 85.34 | 0.15 |
