# Supplementary material for: Classification Models for COVID-19 Test Prioritization in Brazil: Machine Learning Approach
Source: J Med Internet Res. 2021 Apr 8;23(4):e27293. doi: 10.2196/27293 (PMC8034680; doi:10.2196/27293)

SUPPLEMENTARY FIGURES

Figure S1. CD diagram showing the results of critical differences between the classification models using the Nemenyi test based on the recall metric and *RT-PCR Unbalanced* dataset.


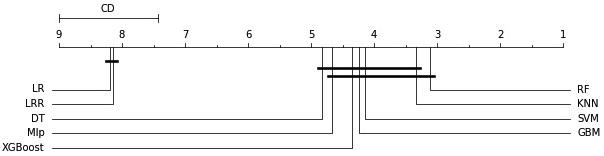


Figure S2. CD diagram showing the results of critical differences between the classification models using the Nemenyi test based on the recall metric and *RT-PCR Balanced* dataset.


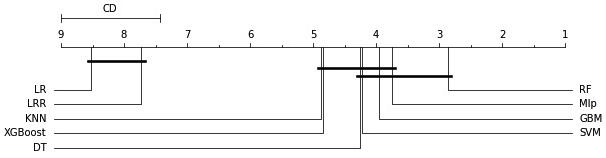


Figure S3. CD diagram showing the results of critical differences between the classification models using the Nemenyi test based on the recall metric and *Rapid Unbalanced* dataset.


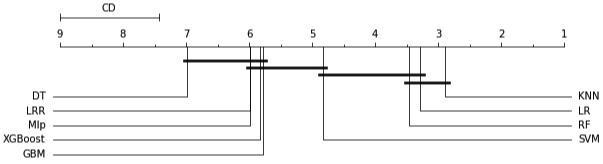


Figure S4. CD diagram showing the results of critical differences between the classification models using the Nemenyi test based on the recall metric and *Rapid Balanced* dataset.


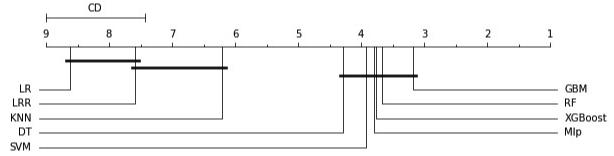


Figure S5. CD diagram showing the results of critical differences between the classification models using the Nemenyi test based on the recall metric and *Both Unbalanced* dataset.


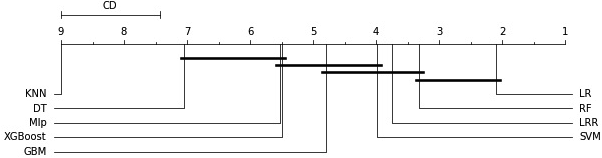


Figure S6. CD diagram showing the results of critical differences between the classification models using the Nemenyi test based on the recall metric and *Both Balanced* dataset.


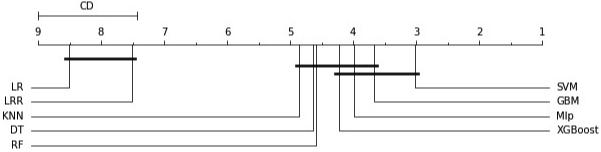

Supplement: Multimedia Appendix 2 [file jmir_v23i4e27293_app2.docx]
