# Supplementary material for: Classification Models for COVID-19 Test Prioritization in Brazil: Machine Learning Approach
Source: J Med Internet Res. 2021 Apr 8;23(4):e27293. doi: 10.2196/27293 (PMC8034680; doi:10.2196/27293)
Supplement: Multimedia Appendix 3 [file jmir_v23i4e27293_app3.docx]

SUPPLEMENTARY TABLES

Table S1. The five most significant features from experimental results for COVID-19 test prioritization using the classification models with highest performances and unbalanced pre-processed datasets.

| Datasets and Models | Top One | Top Two | Top Three | Top Four | Top Five |
| --- | --- | --- | --- | --- | --- |
| **RT-PCR Unbalanced** |  |  |  |  |  |
| MLP | Fever | Gender | Sore Throat | Dyspnea | Cough |
| GBM | Fever | Gender | Sore Throat | Dyspnea | Cough |
| RF | Fever | Gender | Sore Throat | Cough | Dyspnea |
| DT | Fever | Gender | Sore Throat | Dyspnea | Cough |
| XGBoost | Fever | Gender | Cough | Dyspnea | Sore Throat |
| SVM | Fever | Gender | Sore Throat | Dyspnea | Cough |
| **Rapid Unbalanced** |  |  |  |  |  |
| MLP | Dyspnea | Olfactory Disorders | Fever | Gender | Cough |
| GBM | Dyspnea | Olfactory Disorders | Fever | Gender | Cough |
| RF | Dyspnea | Olfactory disorders | Fever | Gender | Cough |
| DT | Dyspnea | Olfactory Disorders | Fever | Gender | Cough |
| XGBoost | Dyspnea | Olfactory Disorders | Fever | Gender | Cough |
| SVM | Dyspnea | Olfactory Disorders | Fever | Gender | Cough |
| **Both Unbalanced** |  |  |  |  |  |
| MLP | Dyspnea | Fever | Gender | Olfactory Disorders | Cough |
| GBM | Dyspnea | Fever | Olfactory Disorders | Gender | Cough |
| RF | Dyspnea | Fever | Gender | Olfactory Disorders | Cough |
| DT | Dyspnea | Fever | Olfactory Disorders | Gender | Cough |
| XGBoost | Dyspnea | Fever | Olfactory Disorders | Gender | Cough |
| SVM | Dyspnea | Fever | Gender | Olfactory Disorders | Cough |

Table S2. The standard deviation and the mean of importance for each feature for the decision-tree-based classification models using the unbalanced pre-processed datasets.

| Datasets and Features | GBM | DT | RF | XGBoost |
| --- | --- | --- | --- | --- |
| **RT-PCR Unbalanced** |  |  |  |  |
| Gender, mean (SD) | 0.211 (0.010) | 0.215 (0.010) | 0.214 (0.009) | 0.206 (0.009) |
| Health Professional, mean (SD) | 0.040 (0.004) | 0.041 (0.004) | 0.047 (0.004) | 0.044 (0.004) |
| Fever, mean (SD) | 0.248 (0.009) | 0.253 (0.008) | 0.251 (0.009) | 0.217 (0.008) |
| Sore Throat, mean (SD) | 0.085 (0.005) | 0.090 (0.005) | 0.089 (0.006) | 0.075 (0.005) |
| Dyspnea, mean (SD) | 0.079 (0.007) | 0.083 (0.007) | 0.081 (0.006) | 0.093 (0.007) |
| Olfactory Disorders, mean (SD) | 0.013 (0.003) | 0.023 (0.003) | 0.020 (0.003) | 0.016 (0.003) |
| Cough, mean (SD) | 0.075 (0.005) | 0.081 (0.005) | 0.082 (0.005) | 0.105 (0.007) |
| Coryza, mean (SD) | 0.022 (0.003) | 0.023 (0.003) | 0.021 (0.003) | 0.013 (0.003) |
| Taste Disorders, mean (SD) | 0.032 (0.003) | 0.032 (0.003) | 0.021 (0.003) | 0.032 (0.003) |
| Headache, mean (SD) | 0.023 (0.003) | 0.025 (0.003) | 0.023 (0.003) | 0.019 (0.003) |
| **Rapid Unbalanced** |  |  |  |  |
| Gender, mean (SD) | 0.025 (0.001) | 0.025 (0.001) | 0.025 (0.001) | 0.024 (0.001) |
| Health Professional, mean (SD) | 0.008 (0.001) | 0.010 (0.001) | 0.008 (0.001) | 0.008 (0.001) |
| Fever, mean (SD) | 0.030 (0.002) | 0.030 (0.002) | 0.028 (0.001) | 0.029 (0.001) |
| Sore Throat, mean (SD) | 0.015 (0.001) | 0.015 (0.001) | 0.015 (0.001) | 0.014 (0.001) |
| Dyspnea, mean (SD) | 0.035 (0.002) | 0.038 (0.002) | 0.034 (0.001) | 0.034 (0.002) |
| Olfactory Disorders, mean (SD) | 0.031 (0.001) | 0.033 (0.001) | 0.029 (0.001) | 0.029 (0.001) |
| Cough, mean (SD) | 0.021 (0.001) | 0.021 (0.001) | 0.020 (0.001) | 0.020 (0.001) |
| Coryza, mean (SD) | 0.012 (0.001) | 0.013 (0.001) | 0.012 (0.001) | 0.011 (0.001) |
| Taste Disorders, mean (SD) | 0.016 (0.001) | 0.016 (0.001) | 0.015 (0.001) | 0.015 (0.001) |
| Headache, mean (SD) | 0.015 (0.001) | 0.016 (0.001) | 0.016 (0.001) | 0.015 (0.001) |
| **Both Unbalanced** |  |  |  |  |
| Gender, mean (SD) | 0.024 (0.001) | 0.026 (0.001) | 0.027 (0.001) | 0.025 (0.001) |
| Health Professional, mean (SD) | 0.012 (0.001) | 0.013 (0.001) | 0.014 (0.001) | 0.013 (0.001) |
| Fever, mean (SD) | 0.033 (0.002) | 0.034 (0.001) | 0.034 (0.001) | 0.033 (0.002) |
| Sore Throat, mean (SD) | 0.016 (0.001) | 0.016 (0.001) | 0.017 (0.001) | 0.017 (0.001) |
| Dyspnea, mean (SD) | 0.035 (0.001) | 0.036 (0.002) | 0.035 (0.001) | 0.035 (0.001) |
| Olfactory Disorders, mean (SD) | 0.027 (0.001) | 0.026 (0.001) | 0.026 (0.001) | 0.027 (0.001) |
| Cough, mean (SD) | 0.022 (0.001) | 0.023 (0.001) | 0.022 (0.001) | 0.022 (0.001) |
| Coryza, mean (SD) | 0.012 (0.001) | 0.011 (0.001) | 0.012 (0.001) | 0.012 (0.001) |
| Taste Disorders, mean (SD) | 0.017 (0.001) | 0.016 (0.001) | 0.016 (0.001) | 0.017 (0.001) |
| Headache, mean (SD) | 0.013 (0.001) | 0.014 (0.001) | 0.015 (0.001) | 0.014 (0.001) |

Table S3. The standard deviation and the mean of importance for each feature for the MLP and SVM models and the unbalanced pre-processed datasets.

| Datasets and Features | MLP | SVM |
| --- | --- | --- |
| **RT-PCR Unbalanced** |  |  |
| Gender, mean (SD) | 0.214 (0.010) | 0.212 (0.010) |
| Health Professional, mean (SD) | 0.042 (0.004) | 0.039 (0.004) |
| Fever, mean (SD) | 0.247 (0.009) | 0.248 (0.008) |
| Sore Throat, mean (SD) | 0.089 (0.005) | 0.086 (0.005) |
| Dyspnea, mean (SD) | 0.081 (0.007) | 0.082 (0.007) |
| Olfactory Disorders, mean (SD) | 0.034 (0.003) | 0.019 (0.003) |
| Cough, mean (SD) | 0.081 (0.006) | 0.080 (0.006) |
| Coryza, mean (SD) | 0.026 (0.003) | 0.013 (0.003) |
| Taste Disorders, mean (SD) | 0.032 (0.004) | 0.030 (0.003) |
| Headache, mean (SD) | 0.028 (0.004) | 0.021 (0.003) |
| **Rapid Unbalanced** |  |  |
| Gender, mean (SD) | 0.025 (0.001) | 0.024 (0.001) |
| Health Professional, mean (SD) | 0.010 (0.001) | 0.008 (0.001) |
| Fever, mean (SD) | 0.031 (0.002) | 0.028 (0.001) |
| Sore Throat, mean (SD) | 0.015 (0.001) | 0.014 (0.001) |
| Dyspnea, mean (SD) | 0.036 (0.002) | 0.034 (0.002) |
| Olfactory Disorders, mean (SD) | 0.034 (0.002) | 0.030 (0.001) |
| Cough, mean (SD) | 0.021 (0.001) | 0.020 (0.001) |
| Coryza, mean (SD) | 0.012 (0.001) | 0.012 (0.001) |
| Taste disorders, mean (SD) | 0.021 (0.001) | 0.016 (0.001) |
| Headache, mean (SD) | 0.016 (0.001) | 0.015 (0.001) |
| **Both Unbalanced** |  |  |
| Gender, mean (SD) | 0.027 (0.001) | 0.025 (0.001) |
| Health Professional, mean (SD) | 0.014 (0.001) | 0.012 (0.001) |
| Fever, mean (SD) | 0.034 (0.001) | 0.033 (0.002) |
| Sore Throat, mean (SD) | 0.018 (0.001) | 0.016 (0.001) |
| Dyspnea, mean (SD) | 0.035 (0.001) | 0.034 (0.002) |
| Olfactory Disorders, mean (SD) | 0.027 (0.001) | 0.025 (0.001) |
| Cough, mean (SD) | 0.023 (0.001) | 0.023 (0.001) |
| Coryza, mean (SD) | 0.012 (0.001) | 0.012 (0.001) |
| Taste Disorders, mean (SD) | 0.018 (0.001) | 0.016 (0.001) |
| Headache, mean (SD) | 0.015 (0.001) | 0.013 (0.001) |
