# Supplementary material for: Classification Models for COVID-19 Test Prioritization in Brazil: Machine Learning Approach
Source: J Med Internet Res. 2021 Apr 8;23(4):e27293. doi: 10.2196/27293 (PMC8034680; doi:10.2196/27293)
Supplement: Multimedia Appendix 4 [file jmir_v23i4e27293_app4.docx]

Table. Results of 10-fold cross validation for the voting ensemble classification models using the unbalanced and balanced pre-processed datasets.

| Datasets and Models | Precision % | Accuracy Score % | Recall % | AUROC % | Brier Score |
| --- | --- | --- | --- | --- | --- |
| **RT-PCR Unbalanced** |  |  |  |  |  |
| GBM, DT, RF, and XGBoost | 97.50 | 96.33 | 97.03 | 95.97 | 0.04 |
| MLP, SVM, KNN, LRR, and LR | 97.45 | 96.66 | 97.58 | 96.18 | 0.03 |
| **RT-PCR Balanced** |  |  |  |  |  |
| GBM, DT, RF, and XGBoost | 96.42 | 95.86 | 95.30 | 95.86 | 0.04 |
| MLP, SVM, KNN, LRR, and LR | 96.06 | 95.69 | 95.34 | 95.69 | 0.04 |
| **Rapid Unbalanced** |  |  |  |  |  |
| GBM, DT, RF, and XGBoost | 99.36 | 98.70 | 99.29 | 91.51 | 0.01 |
| MLP, SVM, KNN, LRR, and LR | 99.22 | 98.79 | 99.52 | 89.85 | 0.01 |
| **Rapid Balanced** |  |  |  |  |  |
| GBM, DT, RF, and XGBoost | 97.15 | 95.43 | 93.67 | 95.43 | 0.05 |
| MLP, SVM, KNN, LRR, and LR | 97.68 | 95.52 | 94.29 | 95.52 | 0.04 |
| **Both Unbalanced** |  |  |  |  |  |
| GBM, DT, RF, and XGBoost | 95.39 | 94.83 | 99.18 | 71.34 | 0.05 |
| MLP, SVM, KNN, LRR, and LR | 94.67 | 94.47 | 99.61 | 66.72 | 0.06 |
| **Both Balanced** |  |  |  |  |  |
| GBM, DT, RF, and XGBoost | 94.30 | 89.32 | 83.76 | 89.33 | 0.11 |
| MLP, SVM, KNN, LRR, and LR | 92.74 | 88.85 | 84.36 | 88.85 | 0.11 |
